# Efficient Dye Contaminant Elimination and Simultaneously Electricity Production via a Bi-Doped TiO_2_ Photocatalytic Fuel Cell

**DOI:** 10.3390/nano12020210

**Published:** 2022-01-10

**Authors:** Dong Liu, Chunling Li, Congyue Zhao, Er Nie, Jianqiao Wang, Jun Zhou, Qian Zhao

**Affiliations:** 1 School of Public Health, Xinxiang Medical University, Xinxiang 453003, China; li_chunling163@163.com (C.L.); zcy19990518@163.com (C.Z.); 2 Engineering Research Center for Nanophotonics and Advanced Instrument, Ministry of Education, School of Physics and Electronic Science, East China Normal University, Shanghai 200241, China; wang.jianqiao@huafeng.com (J.W.); ashley_zhou94@163.com (J.Z.)

**Keywords:** Bi-doped TiO_2_, photocatalytic fuel cell, Rhodamine B, photocurrent, chemical oxygen demand

## Abstract

TiO_2_ develops a higher efficiency when doping Bi into it by increasing the visible light absorption and inhibiting the recombination of photogenerated charges. Herein, a highly efficient Bi doped TiO_2_ photoanode was fabricated via a one-step modified sol-gel method and a screen-printing technique for the anode of photocatalytic fuel cell (PFC). A maximum degradation rate of 91.2% of Rhodamine B (RhB) and of 89% after being repeated 5 times with only 2% lost reflected an enhanced PFC performance and demonstrated an excellent stability under visible-light irradiation. The excellent degradation performance was attributed to the enhanced visible-light response and decreased electron-hole recombination rate. Meanwhile, an excellent linear correlation was observed between the efficient photocurrent of PFC and the chemical oxygen demand of solution when RhB is sufficient.

## 1. Introduction

In recent decades, organic pollutants have attracted widespread attention because of their potential hazards to human health and their hardly degradable characteristics by traditional techniques over the past few decades [1,2,3]. Great efforts have been made to address this problem. Highly effective and environmentally friendly photocatalysis technology based on the catalysts, which are capable of generating oxidant species to eliminate organic pollutants and resist photochemical corrosion, are considered as a promising approach [4,5,6,7,8]. Among alternative catalysts, TiO_2_ shows fascinating superiority for its innocuous character, its cost effectiveness and its high stability [9,10]. Nowadays, the main roadblock in practical application is the rapid recombination of photoexcited electron-hole (e^−^-h^+^) and intensive absorption only in the UV range, which is caused by its wide band gap, which results in a low photocatalytic efficiency [11,12]. Hence, it is necessary to develop effective ways to enhance the visible light responsiveness of TiO_2_. In order to overcome the above disadvantages of TiO_2_, extensive efforts have been put into facilitating the visible light absorption and to enhance the charge carrier separation of TiO_2_. Multiple methods, including non-metal doping [13], metal doping [14], dye sensitization [15] and surface modification [16], have been applied to modify TiO_2_. Among these methods, doping with metal elements is an effective strategy to adjust the internal electronic, expand the light absorption range and reduce the bandgap, which benefits from the new e-states in the band gap and the reduced charge recombination in the photocatalytic process [17]. Transition metal element doping, such as Fe, Co and Ni, could put new d-orbital electronics into TiO_2_, resulting in a charge-transfer transition between the metal elements and the conduction band (CB) or the valence band (VB) of TiO_2_. This kind of dopant can change the electron configuration of TiO_2_ and enhance its photocatalytic activity under visible-light irradiation [18,19,20,21]. Besides transition metal doping, similar phenomena have been reported as well in main group metal doping such as Ga, Sn into TiO_2_ [22,23,24,25]. Different from the original electron configuration of TiO_2_, the new e^−^ states created by metal element doping can enhance the capture of photogenerated electrons and improve the separation of charge carriers [26,27,28].

Among these main group metals, Bi has been demonstrated to be an efficient metal element to improve the photocatalytic activity of TiO_2_ [29,30]. Researchers have endeavored to develop Bi-doped TiO_2_ in recent years. These materials have been put into practical applications, such as organic contaminant elimination [31], hydrogen production [32], and in solar cells [33] and so on. Xu et al. [34] synthesized Bi-doped TiO_2_ nanofibers by electrospinning techniques to modify TiO_2_. It was found that Bi-doping could greatly enhance the photoactivity of Rhodamine B (RhB) degradation. The high photocatalytic performance was attributed to the visible light absorption. Ali et al. [35] prepared Bi-doped TiO_2_ photocatalysts successfully by electrochemical anodization. Fewer charge recombination, and a greater generation of superoxide radicals and hydroxyl radicals led to a higher phenol degradation. Based on these methods, Bi doping was employed to enhance the visible-light absorption and to prevent the recombination of e^−^-h^+^ pairs in TiO_2_, which showed excellent photocatalytic activity for the degradation of organic contaminants.

In the photocatalytic process, the traditional slurry-type reactor, in which separation and reuse of catalysts is difficult, had to restrict the actual application [36]. It is a valid solution to immobilize photocatalysts on electrode substrates in order to constitute a photocatalytic fuel cell (PFC). Nowadays, PFCs have been used as an integrated system for electricity production and simultaneously for organic contaminant elimination [37]. Currently, researchers have facilitated the photocatalysts to the photoanodes of PFC and have obtained a stable and efficient system where the degradation performance of PFC is mainly up to the photoanodes [38,39,40,41]. Therefore, it has great prospects to combine the highly efficient visible light-responsive Bi-doped TiO_2_ photoanodes with stable PFC systems together. 

In this work, Bi-TiO_2_ was prepared through a one-step sol-gel method. The possible formation mechanism of Bi-TiO_2_ composites was discussed. The PFC based on Bi-TiO_2_ photoanode was constructed to achieve immobilized photocatalysts, in which RhB was chosen to assess the performance of organic degradation. The chemical oxygen demand (COD) of RhB was detected by standard methods and was calculated by recording the electrons transferred simultaneously to study the photocurrent.

## 2. Materials and Methods

### 2.1. Preparation of Catalysts

All of the reagents were up to analytical grade and were purchased from Sinopharm Chemical Reagent Co. Ltd. (Shanghai, China). Bi-TiO_2_ was synthesized through a one-step sol-gel process. Briefly, 5 mL of tetrabutyl titanate was dissolved in 25 mL of ethanol and stirred for 1 h, indicated as solution A. Meanwhile, a certain amount of Bi(NO_3_)_3_·5H_2_O, 0.5 mL of diacetone and 10 mL of acetic acid was added subsequently in 2 mL of ultrapure water stirring for 1 h, denoted as solution B. The solution B was added into solution A drop by drop under sharp stirring. The mixture underwent a continuous stir until it became a transparent sol due to hydrolysis of tetrabutyl titanate. All the operations above were accomplished at room temperature. After being aged for 12 h, the resulting sol was held at 80 °C for another 12 h. Finally, the Bi-TiO_2_ was obtained after calcination at 400 °C for 2 h in air atmosphere. The as-prepared Bi-TiO_2_ samples with 1–5 at.% Bi were named BT-1, BT-2, BT-3, BT-4 and BT-5. The pure TiO_2_ was also prepared for comparison in such a method without Bi(NO_3_)_3_·5H_2_O. The as-synthesized Bi-TiO_2_ was loaded onto the electrode substrate by a screen-printing method, as reported in our previous work [22].

### 2.2. Characterization

The morphologies and structures of catalysts were observed by field-emission scanning electron microscopy (FESEM, Hitachi S-4800, Tokyo, Japan) and energy-dispersive X-ray spectroscopy (EDX). The chemical compositions were performed by X-ray photoelectron spectroscopy (XPS, Axis Ultra, Kratos Analytical, Manchester, UK) with a monochromatic Al Kα X-ray source. The crystal phase structures were determined by powder X-ray diffraction (XRD) with an X-Ray diffractometer (PRO PW 3040/60, V 30 kV, I = 25 mA, PANalytical, EA Almelo, The Netherlands) with Cu Kα radiation. UV-Vis spectra of as-prepared samples was achieved by a UV-Vis spectrophotometer (Hitachi U-3900, Tokyo, Japan) with BaSO_4_ as a reflectance standard. Photocurrent measurement of the photoanodes were tested on an electrochemical workstation (AUTOLAB PGSTAT302N, Metrohm Autolab, The Netherlands) with a solar simulator (100 mW cm^−2^). The electrode used a three-electrode configuration with Bi-TiO_2_ photoelectrodes as a working electrode, Pt electrode as the counter electrode, and a standard calomel electrode as the reference electrode, and the electrolyte was 0.1 M Na_2_SO_4_ in aqueous solution. The photoluminescence (PL) spectra was obtained by fluorescence spectrophotometer (Fluoromax-4, HORIBA Jobin Yvon, Kyoto, Japan) with an excitation wavelength of 300 nm. Electrons transferred from the external circuit of PFC was carried out on an electrochemical workstation (ZF-100, Zhengfang, Shanghai, China). The total organic carbon (TOC) was detected using a TOC analyzer (TOC-L CPN, Shimadzu, Kyoto, Japan). The COD of the treated solution was analyzed using a COD analyzer (DR1010 and DRB200, HACH, Loveland, CO, USA).

### 2.3. PFC Performance

The degradation experiment was carried out in the PFC based on immobilized Bi-TiO_2_ photoanode and Pt cathode. The distance between the two electrodes was set at 1.0 cm. The PFC was working via a photocatalytic reactor (Shanghai Bilon Co., Ltd., Shanghai, China), in which the scale of the filter (λ > 420 nm) was 30 mm × 100 mm and the interval between quartz tube and 450 W metal halide lamp was 60 mm. Na_2_SO_4_ (0.01 mol) was added to 80 mL aqueous solutions of RhB (10 mg L^−1^) to enhance the conductivity. The adsorption-desorption equilibrium of the PFC system was reached after 1 h magnetic stir in the dark. The mixture was being stirred and irradiated under the visible light with ambient temperature and pressure. 2 mL of the reaction solution was taken to detect by UV-vis spectrophotometer (Hitachi U-3900, Tokyo, Japan) with the corresponding wavelengths range 400 nm to 600 nm, and COD analyzer (Hach-COD, Loveland, CO, USA) after digestion in 165 °C for 15 min.

## 3. Results

### 3.1. Structural and Morphological Characteristics

**Figure 1:**
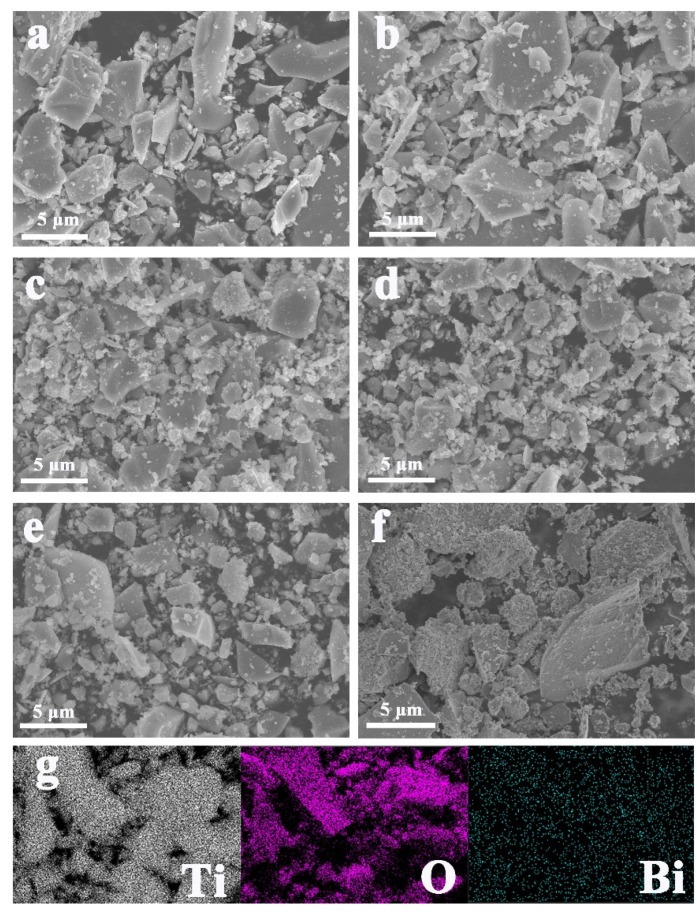
SEM images of (**a**) pure TiO_2_, (**b**) BT-1, (**c**) BT-2, (**d**) BT-3, (**e**) BT-4, (**f**) BT-5; (**g**) Elemental mapping images of BT-3.

Figure 1 displayed the FESEM images of pure TiO_2_, BT-1, BT-2, BT-3, BT-4 and BT-5. Obviously, all the samples presented a similarly irregular nanostructure, indicating little effect on the morphology of TiO_2_ with Bi dopant. In addition, according to the elemental mapping images of BT-3, we confirmed the presence of Bi, Ti and O elements, where the Bi elements were evenly distributed in the catalysts. The above results indicated that the Bi elements were doped into TiO_2_ successfully.

**Figure 2:**
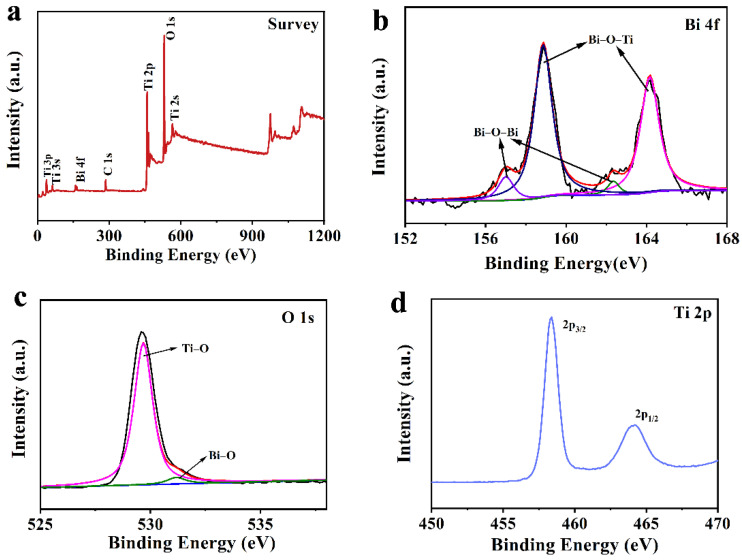
XPS patterns of BT-3 (**a**) survey spectrum; (**b**) Bi 4f spectrum; (**c**) O 1s Spectrum; (**d**) Ti 2p spectrum.

The chemical composition was further explored by XPS. As shown in Figure 2a, Bi, Ti, O and C elements evidently existed. The C 1s peak ought to be induced by the environmental carbon element. Two fitted peaks located in the high-resolution spectrum of Bi 4f demonstrated that there were two forms of Bi in BT-3 (Figure 2b). The Bi–O–Ti bonds might be attributed to the binding energy at 158.9 eV and 164.2 eV, indicating that a part of Ti atoms were replaced by Bi atoms and the high oxidation state of Bi (Bi^(3+δ)+^) was generated as the hydrolysis process proceeded. The Bi–O–Bi bonds corresponded to the binding energy centered at 157.0 eV and 162.3 eV [6,34,42]. Two fitted peaks of O 1s spectrum at 529.7 and 531.2 eV (Figure 2c) corresponded to Bi–O, and Ti–O bonds. The Ti 2p_3/2_ and Ti 2p_1/2_ peaks at 458.3 and 464.1 eV confirmed the existence of Ti^4+^–O bonds in BT-3 (Figure 2d) [27].

**Figure 3:**
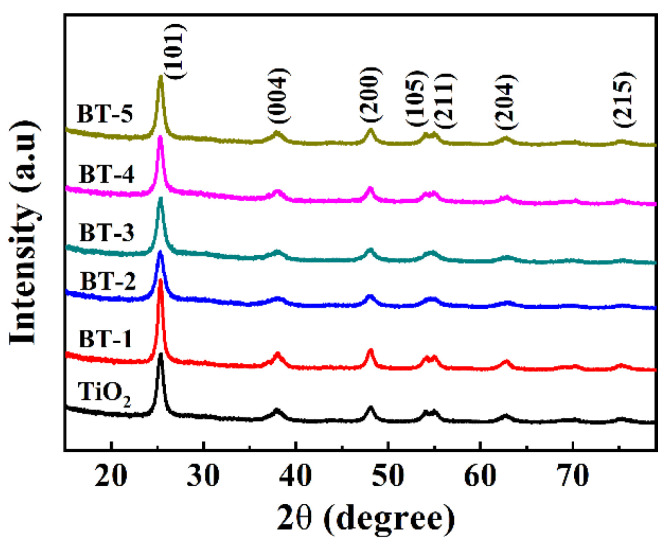
XRD patterns of as-prepared samples.

The XRD patterns exhibited a high crystallinity degree and the same diffraction of samples (Figure 3). All of them presented peaks at 25.8°, 37.8°, 48.0°, 53.9° and 55.1°, indexed to (101), (004), (200), (105), (211) and (204) crystal planes (JCPDS 21-1272), and the anatase TiO_2_ phase was responsible for all these peaks without the other crystalline phases. In terms of diffraction peaks from the XRD analysis, the dopant was not considered to bring in a new phase to TiO_2_ [24,29]. The XRD patterns around 25.8° showed that the (101) diffraction peaks of Bi-TiO_2_ shifted to the left, further confirming the existence of Bi in the lattice of TiO_2_ [43,44]. The XRD results evidently illustrated Bi doping into TiO_2_ successfully without any noisy or unexpected diffraction peak, showing a high purity of the samples obtained from the simple sol-gel synthesis process.

### 3.2. Spectral and Photoelectric Properties

**Figure 4:**
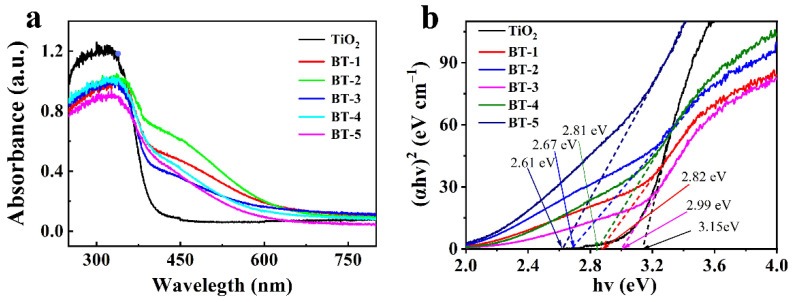
(**a**) UV-Vis diffuse reflectance spectra of the samples; (**b**) the bandgap values transformed by a Kubelka-Munk function.

Figure 4a displayed the UV-Vis diffuse reflectance spectra. A red-shift appeared in the absorption curves of Bi-TiO_2_ compared with pure TiO_2_, meaning that the ability of doped catalysts to absorb visible light ranging from 400 to 600 nm was significantly enhanced. The addition of Bi allowed the coexistence of the Bi electrons and the CB electrons of TiO_2_ and the carrier charge-transfer transitions in between [45]. According to the Kubelka–Munk Function (Equation (1)), we transformed the UV-Vis diffuse reflection absorption spectra of as-prepared samples to calculate the band gap size of theirs:

(*α*h*ν*)^2^
*=* A(h*ν −* Eg) (1)

In this equation, “*α*” and “h*ν*” are referred to as absorption coefficient and photon energy while “A” and “Eg” on the right side of the equation are a constant and the energy of the band gaps respectively. When (*α*h*ν*)^2^ = 0, “Eg” is also regarded as the intercept of the curve. The band gap energies of TiO_2_ BT-1, BT-2, BT-3, BT-4 and BT-5 were 3.15, 2.82, 2.67, 2.99, 2.81 and 2.61 eV as shown in Figure 4b. The results indicated that Bi doping noticeably shrank the TiO_2_ band gap and enhanced the absorption in the visible region.

**Figure 5:**
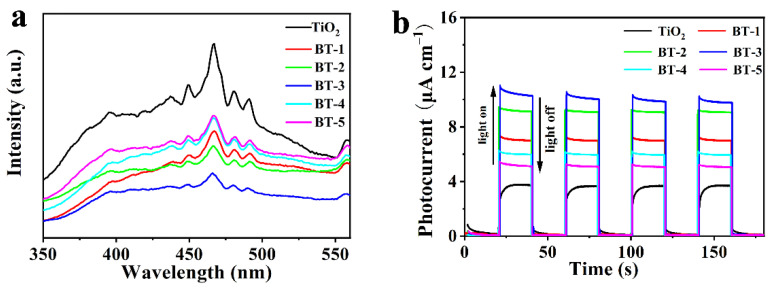
(**a**) PL spectra of as-synthesized samples; (**b**) photocurrent of as-prepared photoanodes.

The photoelectrochemical measurements was reflected by PL spectra to explore the separation and recombination of photocarriers. As shown in Figure 5a, Bi-TiO_2_ performed a lower emission peak than pure TiO_2_, demonstrating that Bi could suppress the recombination of photocarriers. Meanwhile, BT-3 had the lowest emission peak among all the samples, which meant the lowest recombination rate of e^−^ and h^+^, in line with the photodegradation test shown in Figure S1. The formation of Bi^(3+*δ*)+^ could possibly account for this phenomenon because it was reported to be able to enhance the separation of e^+^ and h^+^ [42]. Bi was confirmed in its high oxidation state by the XPS analysis in the samples, as Bi^(3+*δ*)+^ would capture these photo-generated electrons and then be reduced to a lower energy state, such as Bi^3+^.

The intermittent-irradiation photocurrent could reflect the separation of e^−^-h^+^. As shown in Figure 5b, the I-t curves of the TiO_2_ and BT-1 to BT-5 photoanodes with four on–off cycles verified the sensitive response of the photoanodes. The photocurrents were efficiently enhanced by Bi doping. The maximum value (10.2 μA) was found in the BT-3, which indicated that the isolation of photoexcited charges could be accelerated with the addition of Bi^(3+*δ*)+^ so as to generate more carriers. However, the effect was diminished as the percentage of Bi further increased after 3%. The factor that excessive Bi dopant created the new centers of photogenerated e^−^-h^+^ could account for the result [45,46].

### 3.3. Effect of Bi Content on the PFC Performance

**Figure 6:**
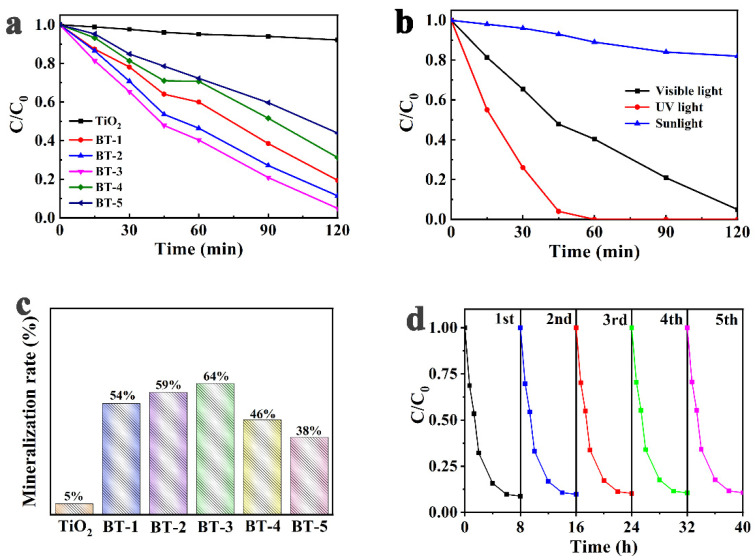
(**a**) PFC degradation of RhB of as-prepared photoanodes under visible light irradiation; (**b**) PFC degradation of RhB by BT-3 sample under visible light, UV light and sunlight irradiation; (**c**) TOC of RhB in the PFC using the as-prepared samples under visible light irradiation; (**d**) repeatability test of BT-3 photoanode for 5 times PFC process.

The degradation of RhB under visible-light by TiO_2_, BT-1, BT-2 BT-3 BT-4 and BT-5 PFC were assessed, as shown in Figure 6a. The variation of the maximum absorbance (A/A_0_) was recorded in the RhB absorption spectra at a certain time interval to reflect the variation of normalized temporal concentration (C/C_0_) of RhB during the PFC process.

The degradation of RhB was satisfied with the first-order kinetic equation. The degradation rate constants of TiO_2_ BT-1, BT-2, BT-3, BT-4 and BT-5 were shown in Table S1. The degradation rate constant of TiO_2_ was located on 1.50 × 10^−3^ min^−1^, while the BT-1, BT-2, BT-3, BT-4 and BT-5 were 3.70, 4.20, 5.20, 2.60 and 1.80 × 10^−3^ min^−1^, respectively. It can be seen that the degradation rate constant depended on the Bi doping concentration, which increased to the highest in BT-3 photoanode, about 3.5 times higher than pure TiO_2_. All of the Bi-TiO_2_ photoanodes tested in this work had a better photocatalytic performance than pure TiO_2_ in terms of degradation rate. The increase in the visible-light absorption and the decrease in electron-hole pair recombination might be responsible for the enhancement of photocatalytic activity, which was in accord with UV-Vis absorption and PL spectra. A further increase in Bi content in BT-4 and BT-5 did not lead to the synchronous increase in performance, but a decrease on the contrary, and the creation of new recombination centers might account for the phenomenon.

The degradation rate of RhB depended on the Bi doping concentration, which increased from 49% of pure TiO_2_ to 83% of BT-1 in 120 min and reached a maximum rate of 91% for BT-3. The BT-3 had the best efficiency in both powder and photoanode as shown in Figure S1, which indicated that the photoanodes prepared by the screen-printing method did not change the photoactivity of Bi-TiO_2_. The degradation experiments under different photosources (visible light, UV light and sunlight) were also performed to further investigate the catalytic activity of the BT samples. As shown in Figure 6b, the highest degradation rate was obtained under UV light, and 100% of RhB were removed within 60 min of PFC reaction. However, only 17.8% of RhB were degraded under direct sunlight irradiation due to the low light density of sunlight. The mineralization degree of organics was also evaluated by the TOC removal of RhB as shown in Figure 6c. The BT-3 exhibited the maximum removal rate of TOC (64%), yet only 5% of organics were mineralized by the pure TiO_2_ in comparison. These results indicated a significant enhancement of TiO_2_ in the mineralization capacity under visible light irradiation by Bi doping.

The Bi-TiO_2_ photoanodes demonstrated a reliable repeatability, as shown in Figure 6d. After five-time repetitions, the degradation rate dropped only 2%, to 89%, and no obvious abscission was observed on the surface of photoanodes. The photoanodes were effective for the separation and recycling of photocatalysts. The SEM image and XRD pattern of BT-3 after the photocatalytic reaction were also investigated to further evaluate the stability of the catalyst. As shown in Figures S2 and S3, there was no significant change in the its microtopography and crystal form before and after the photocatalytic experiment, which suggested that the BT catalysts exhibited good stability. In addition, to elucidate the roles of the active species in the PFC reaction, quenching experiments were conducted. As shown in Figure S4, isopropanol (IPA), benzoquinone (BQ) and EDTA-Na_2_ were selected to capture hydroxyl radicals, superoxide radicals and holes, respectively. Both IPA and EDTA-Na_2_ showed obvious effects in inhibiting the degradation rate of RhB. In contrast, negligible restraint was observed after the addition of BQ, indicating that hydroxyl radicals and holes played a paramount role in the degradation of RhB, and superoxide radicals showed weak contributions.

### 3.4. Photocurrent Properties for Determination of COD

Although the efficiency of energy transformation remains to be improved, the photocurrent could be used to assess water quality due to its correlation with organic pollution conditions [47,48,49]. Therefore, the COD value, representing the total pollution load of most wastewater discharges, can be detected by monitoring the photocurrent produced during the oxidation of organic compounds under photocatalytic conditions [50]. The oxygen evolution reaction in the PFC system depended on water splitting and other conditions, and could be recorded as blank when there is no organic compound fuel such as RhB in aqueous solutions. Thus, the organic oxidation electricity generated in PFC with RhB as a fuel could equal the total current minus the blank current [51].

**Figure 7:**
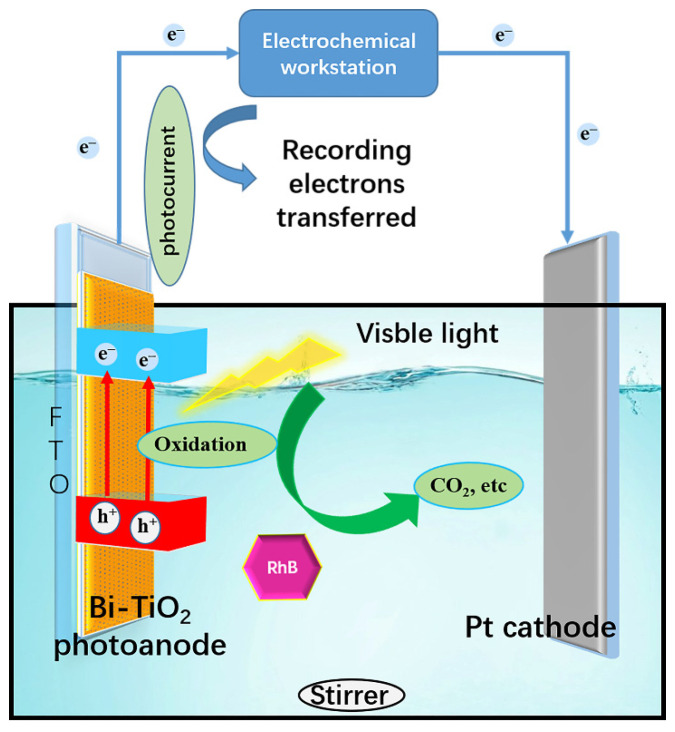
Structure of PFC connecting with the electrochemical workstation.

As shown in Figure 7, the PFC system and electrochemical workstation were connected to record the photocurrent and the net charge for organic oxidation transferred is calculated by Equation (2); the results are shown in Figure S5:

(2) $$Q_{net}=\int_{0}^{t}{(I}_{\mathrm{total}}-{\;I}_{\mathrm{blank}})\mathrm{dt}\;$$

**Figure 8:**
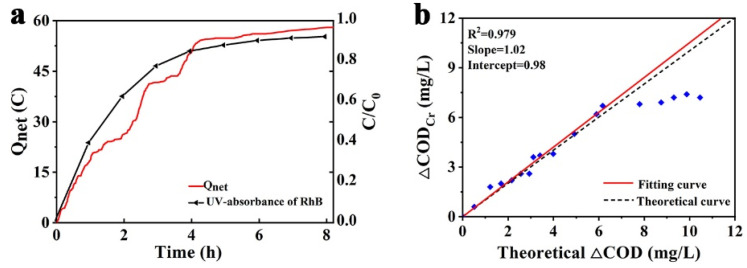
(**a**) Net charge transferred and UV-Vis absorbance of RhB with irradiation of PFC; (**b**) the Correlations between TECOD with actual COD.

The relationship between the net charge transferred with the irradiation of the PFC system and the UV-Vis absorbance of RhB was shown in Figure 8a. Experimental results showed that the charge transferred through the external circuit drops sharply with the concentration of fuel decline. The performance of the PFC was mainly controlled by the activation of the photoelectron, which was limited by the poor mass transfer at the photoanode due to lower concentration of fuel [38]. According to the definition of COD and Faraday’s Law, the measured *Q_net_* value could be converted into an equivalent oxygen demand. The theoretical equivalent COD (TECOD) can therefore be represented as [47]:

(3) $$\mathrm{TECOD}=\frac{Q_{\mathrm{net}}}{4\mathrm{FV}}\;\times \;32\;\times \;1000\;\;(\mathrm{mg}\;L^{-1})$$

where F and V are the faraday constant and the volume of aqueous solution, and 4 represented that 4 mol *Q_net_* is equivalent to 1mol O_2_ under optimal conditions. 

The value of TECOD, derived from Equation (3) with *Q_net_* obtained before, was compared with the actual COD in the aqueous solution, measured by the standard method. As shown in Figure 8b, the TECOD exhibited an excellent correlation, with actual COD in the first 180 min. The slope of the fitting curve was 1.02, which indicates that the error was about 5% and the TECOD was slightly over, and was considered as the analytical error. In this interval, the PFC could still reflect the actual COD through *Q_net_* accurately. However, as the process continued, the actual COD did not drop in sync with the decline of RhB. This might be explained as follows: under a lower fuel concentration, the mass transport issue became more significant and the recombination of excited electron-hole pairs became more rapid at a lower cell voltage as well [38].

## 4. Conclusions

In summary, Bi-TiO_2_ catalysts were successfully synthesized via a simple sol-gel method, endowing TiO_2_ with enhanced visible light absorption and reduced charge recombination. The PFC based on the as-synthesized Bi-TiO_2_ photoanodes was constituted via a simple screen-painting method, and exhibited an effective elimination capability of RhB. Furthermore, the photocurrent was studied by comparing the theoretical COD value with the actual value. The electron transfer was recorded to calculate the theoretical COD, which turned out to correlate with actual COD within, due to the enhanced mass transfer. These results indicated that the Bi-doped TiO_2_ photocatalytic fuel cell had the characteristics of the efficient dye contaminant elimination and a simultaneous electricity production.

## Data Availability

The data presented in this study are available on request from the corresponding authors.

## Supplementary Materials

The following are available online at https://www.mdpi.com/article/10.3390/nano12020210/s1, Figure S1: Photocatalytic degradation of RhB by TiO_2_ and BT powers under visible light irradiation, Figure S2: SEM image of BT-3 after the photocatalytic reaction, Figure S3: XRD pattern of BT-3 after the photocatalytic reaction, Figure S4: PFC degradation of RhB with existence of scavengers, Figure S5: The Q_total_ and Q_blank_ of PFC process, Table S1: The kinetic equation of TiO_2_ and 1–5% Bi-TiO_2_ PFC.
