# Supplementary material for: Efficient Dye Contaminant Elimination and Simultaneously Electricity Production via a Bi-Doped TiO2 Photocatalytic Fuel Cell
Source: Nanomaterials (Basel). 2022 Jan 10;12(2):210. doi: 10.3390/nano12020210 (PMC8778080; doi:10.3390/nano12020210)
Supplement: Supplementary file 1 [file nanomaterials-12-00210-s001.zip › nanomaterials-1535439-supplementary.pdf]

Supplementary material

Efficient Dye Contaminant Elimination and Simultaneously Electricity Production via a Bi-Doped TiO_2 Photocatalytic Fuel Cell

Dong Liu [1,2,*], Chunling Li [1], Congyue Zhao [1], Er Nie [2,*], Jianqiao Wang [2], Jun Zhou [2] and Qian Zhao [1,*]

[1] School of Public Health, Xinxiang Medical University, Xinxiang 453003, China; li_chunling163@163.com (C.L.); zcy19990518@163.com (C.Z.);
[2] Engineering Research Center for Nanophotonics and Advanced Instrument, Ministry of Education, School of Physics and Electronic Science, East China Normal University, Shanghai 200241, China; wang.jianqiao@huafeng.com (J.W.); ashley_zhou94@163.com (J.Z.)
* Correspondence: liudong081@163.com (D.L.); enie@phy.ecnu.edu.cn (E.N.); zhqzhaoqian@126.com (Q.Z.); Tel.: +86-373-3831063 (D.L.)

Photocatalytic degradation of RhB by TiO_2, BT-1, BT-2 BT-3 BT-4 and BT-5 powers were performed under visible light irradiation, as shown in Figure S1. It is observed that BT-3 exhibited the highest photocatalytic performance. The BT-3 has the best efficiency in both powder and photoanode.

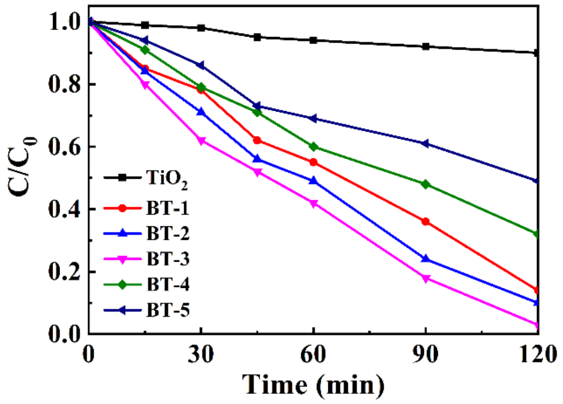

Figure S1. Photocatalytic degradation of RhB by TiO_2 and BT powers under visible light irradiation.

The SEM image and XRD pattern of BT-3 after the photocatalytic reaction were also investigated to further evaluate the stability of the catalyst. As shown in Figure S2 and Figure S3, there was no significant change in the its microtopography and crystal form before and after the photocatalytic experiment, which suggested that the BT catalysts exhibited good stability.

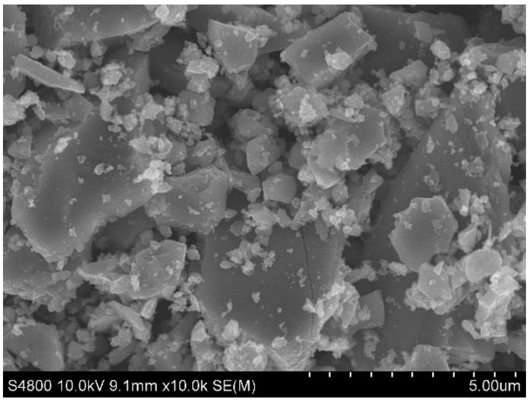

Figure S2. SEM image of BT-3 after the photocatalytic reaction.

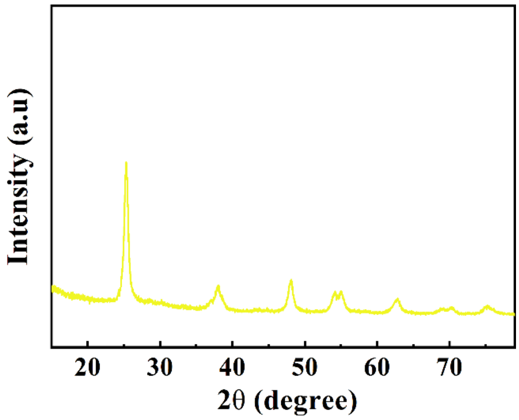

Figure S3. XRD pattern of BT-3 after the photocatalytic reaction.

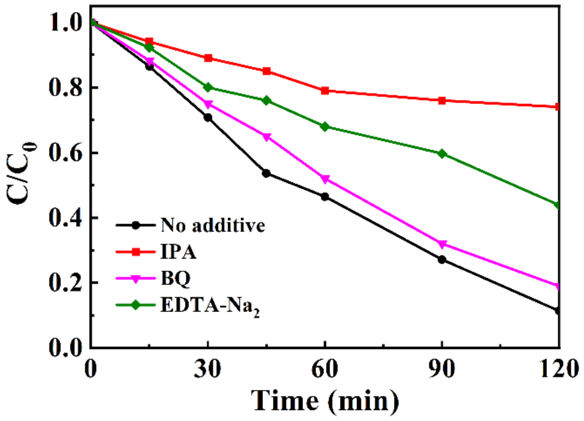

Figure S4. PFC degradation of RhB with existence of scavengers.

The electron transferrd was recorded dircetly by ZF-100 electrochemical workstations. The charges transfer were regard as Q_{total} when 10 mg L^{-1} of RhB aqueous solutions was obtained and Q_{blank} without any fuel. The net charge was obtained by Q_{total} minus Q_{blank}.

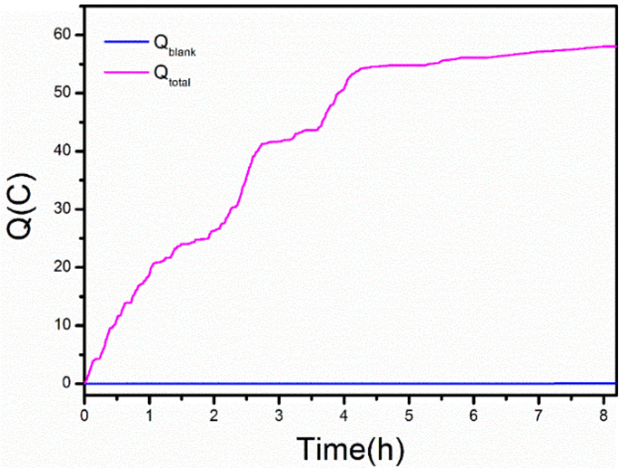

Figure S5. The Q_{total} and Q_{blank} of PFC process.

The PFC degradation of RhB was fitted by the first-order kinetic equation as shown in the Table S1.

Table S1. The kinetic equation of TiO_2 and 1–5% Bi-TiO_2 PFC

Photoanode	Kinetic Equation	R^2
Pure TiO_2	$-\ln(C/C_0) = 0.0015t - 0.0145$	0.9953
BT-1	$-\ln(C/C_0) = 0.0037t + 0.106$	0.9894
BT-2	$-\ln(C/C_0) = 0.0042t - 0.0022$	0.9988
BT-3	$-\ln(C/C_0) = 0.0052t + 0.2609$	0.9292
BT-4	$-\ln(C/C_0) = 0.0026t - 0.0003$	0.9713
BT-5	$-\ln(C/C_0) = 0.0018t + 0.0097$	0.9891
